# Efficient Green Quasi-Two-Dimensional Perovskite Light-Emitting Diodes Based on Mix-Interlayer

**DOI:** 10.3389/fchem.2021.825822

**Published:** 2022-01-17

**Authors:** Zirong Wang, Fanyuan Meng, Qi Feng, Shengxuan Shi, Langwen Qiu, Guanwei Sun, Zhao Chen, Qingguang Zeng, Weiguo Zhu, Shi-Jian Su

**Affiliations:** ^1^ School of Applied Physics and Materials, Wuyi University, Jiangmen, China; ^2^ State Key Laboratory of Luminescent Materials and Devices, Institute of Polymer Optoelectronic Materials and Devices, South China University of Technology, Guangzhou, China; ^3^ Jiangsu Engineering Laboratory of Light-Electricity-Heat Energy-Converting Materials and Applications, Changzhou University, Changzhou, China

**Keywords:** quasi-two-dimensional perovskites, mix-interlayer, phase compositions, interlayer engineering, perovskite light-emitting diodes

## Abstract

Recently, quasi-two-dimensional (Q-2D) perovskites have received much attention due to their excellent photophysical properties. Phase compositions in Q-2D perovskites have obvious effect on the device performance. Here, efficient green perovskite light-emitting diodes (PeLEDs) were fabricated by employing o-fluorophenylethylammonium bromide (o-F-PEABr) and 2-aminoethanol hydrobromide (EOABr) as the mix-interlayer ligands. Phase compositions are rationally optimized through composition and interlayer engineering. Meanwhile, non-radiative recombination is greatly suppressed by the introduction of mix-interlayer ligands. Thus, green PeLEDs with a peak photoluminescence quantum yield (PLQY) of 81.4%, a narrow full width at half maximum (FWHM) of 19 nm, a maximum current efficiency (CE) of 27.7 cd/A, and a maximum external quantum efficiency (EQE) of 10.4% were realized. The results are expected to offer a feasible method to realize high-efficiency PeLEDs.

## Introduction

Metal-halogen perovskites are emerging as potential candidates for light-emitting diodes due to their high color purity (insensitive to crystal size), high photoluminescence quantum yield (PLQY), facile adjustable photoelectronic properties, and solution processability (Wang et al., 2018a; Miao et al., 2019; Quan et al., 2019; Han et al., 2021). Impressive electroluminescence efficiency of perovskite light-emitting diodes (PeLEDs) has been demonstrated in recent years (Meng et al., 2020; Liu et al., 2021a; Liu et al., 2021b; Hassan et al., 2021; Zhu et al., 2021). Currently, numerous research efforts focused on development of perovskite electroluminescent materials with high PLQY, good film quality, and efficient carrier injection.

Three-dimensional (3D) perovskites with continuous octahedral frameworks generally feature in a weak exciton binding energy (Miyata et al., 2015; Yang et al., 2016). Excitons in 3D perovskites tend to separate into free carriers which induce non-radiative recombination. Q-2D perovskites with a multiple quantum-well structure are another approach to achieve efficient electroluminescence (He et al., 2019; Guo et al., 2021; Jiang et al., 2021; Yang et al., 2021). The multiple quantum-well structure of Q-2D perovskites is beneficial to exciton formation and reducing the possibility of exciton dissociation. In addition, Q-2D perovskites are a strongly bound system due to the strong quantum and dielectric confinement effects (Blancon et al., 2017; Xing et al., 2017; Zhang et al., 2018; Cheng et al., 2020). These effects contribute to improving the exciton binding energy, adjusting the photoelectric properties, and inhibiting the non-radiative recombination. There exists an ultrafast energy transfer process from the large bandgap phases (small n values) to the lowest bandgap phase (larger n values) in Q-2D perovskites, which promises efficient radiative recombination (Liu et al., 2017; Panuganti et al., 2021; Yin et al., 2021). Hence, Q-2D perovskites are considered good candidate materials for efficient PeLEDs. This unique emission behavior of Q-2D perovskites is strongly dependent on the perovskite phase compositions. Up to now, it is still difficult to obtain the Q-2D perovskites with single n phase (n ≥ 2), and the Q-2D perovskite films are usually a mixture with different n phases (Tsai et al., 2018; Yang et al., 2018; Yang et al., 2019). Previous studies have shown that enhanced phase purity has a significant effect on charge transfer between different n phases and eventual device performance (Wang et al., 2018b; Yang et al., 2018; Yu et al., 2019). Organic interlayer ligands in Q-2D perovskites can directly affect the phase compositions (purity). The subtle structure change in organic interlayer ligands might lead to a big difference in perovskite phase compositions. Therefore, selecting suitable organic interlayer ligands to regulate the phase compositions in Q-2D perovskites is expected as a feasible strategy to further improve the device efficiency.

In this work, o-F-PEABr and EOABr were employed as the mix-interlayer ligands to fabricate Q-2D PeLEDs. Phase compositions and energy levels can be effectively regulated by the o-F-PEABr ligand. On combining with the EOABr ligand, the non-radiative recombination channels in these Q-2D perovskites are greatly suppressed. Efficient green PeLEDs based on mix-interlayers were realized with an emission peak at 509 nm, a maximum CE of 27.7 cd A^−1^, and a maximum EQE of 10.4%. The results demonstrated here offer a simple path to realize efficient PeLEDs.

## Results and Discussion

Perovskite precursor solutions were prepared by dissolving o-F-PEABr, lead bromide, and cesium bromide in dimethyl sulfoxide solvent according to the Q-2D perovskite general formula o-F-PEA_2_Cs_m-1_Pb_m_Br_3m+1_ with various m values (the *Experimental section*). Perovskite emitters were prepared by the one-step spin-coating method. To study the effect of phase compositions on PeLED performance, a group of devices with a configuration of ITO (100 nm)/polyvinylcarbazole (PVK):N,N′-bis(4-butylphenyl)-N,N-bis(phenyl)-benzidine (TPD) (30 nm)/perovskite emitters (60 nm)/3,5-Tris(1-phenyl-1H-benzimidazol-2-yl)benzene (TPBI) (35 nm)/LiF (1 nm)/Al (150 nm) were fabricated. PVK:TPD acted as a hole-transporting layer, and TPBI was used as an electron-transporting layer. The perovskite emitters with m = 1, 2, 3, 4 were employed as the light-emitting layers. PeLEDs based on the perovskite emitters with m = 1 were also fabricated; however, device performance could be hardly observed. As previously reported, this should be attributed to their poor film morphology, large hole-injection barrier, and serious traps assisting non-radiative recombination (Era et al., 1994; Xing et al., 2017).

The electroluminescence (EL) performance of these PeLEDs is shown in Supplementary Figure S1 and Supplementary Table S1. All these PeLEDs show green emission from 511 to 502 nm (Supplementary Figure S1A). The EL peaks show a slightly red shift with the m values increased. The m = 3 (o-F-PEA_2_Cs_2_Pb_3_Br_10_) PeLEDs show optimum device performance with a peak luminance of 7,290 cd/m^2^, a maximum CE of 14.2 cd A^−1^ (Supplementary Figure S1C), and a maximum EQE of 5.1% (Supplementary Figure S1D). The turn-on voltage (V_on_) at 3.0 V certifies the carriers were effectively injected into the perovskite emitters (Supplementary Figure S1B), which can be proved by the smaller hole-injection barrier (Supplementary Figure S2). Therefore, the m = 3 perovskite was used as the optimal condition for further study.

As the ultraviolet–visible (UV–Vis) absorption spectra shown in Supplementary Figure S3A, the phase compositions of o-F-PEA_2_Cs_m-1_Pb_m_Br_3m+1_ Q-2D perovskite can be effectively regulated by changing the m values. The single absorption peak at 396 nm of m = 1 (o-F-PEA_2_PbBr_4_) perovskite is attributed to the n = 1 phase. The absorption peaks of m = 2 (o-F-PEA_2_CsPb_2_Br_7_) perovskite at 396, 428, and 456 nm are attributed to n = 1, 2, 3 phases, respectively. The n = 1, 2 phases could be greatly inhibited as the m = 3 (o-F-PEA_2_Cs_2_Pb_3_Br_10_) and 4 (o-F-PEA_2_Cs_3_Pb_4_Br_13_) perovskites. The PLQY of m = 1, 2, 3, 4 is 1.1%, 6.8%, 58.7.1, and 46.8%, respectively.

The morphology of these perovskites was studied through the scanning electron microscopy (SEM) test. As shown in Supplementary Figure S4, the m = 1 perovskite films show a rough and wrinkle morphology. The film quality showed notable improvement with the m values increased from 2, 3 to 4. Thus, the improved PeLED performance of m = 3 perovskites comes from the combination of improved phase purity, higher film quality, efficient carrier injection, and higher PLQY.

**FIGURE 1:**
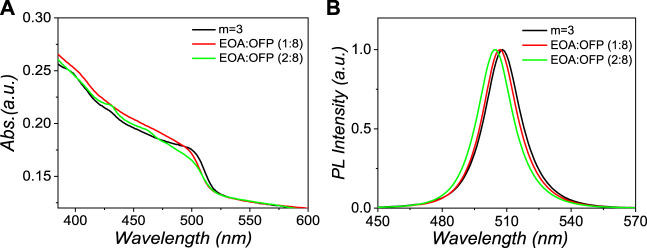
**(A)** UV–Vis absorption spectra and **(B)** PL spectra of the corresponding Q-2D perovskite films with different interlayers.

The employment of mix-interlayer ligands with synergistic effects is an effective way to improve the device performance. Here, mix-interlayer perovskites were fabricated by introducing the EOABr ligand with the general formula of (EOA _x_ o-F-PEA _y_)_2_Cs_2_Pb_3_Br_10_ (x + y = 1). The phase compositions were further engineered by tuning the mix-interlayer ligand molar ratio with x:y = 1:8, 2:8 toward efficient quasi-2D perovskites. UV–Vis absorption and photoluminescence (PL) spectra of these samples are exhibited in Figure 1A; the m = 3 sample is also included for comparison. The m = 3 and 1:8 samples show weak excitonic absorption peaks of low-n phases (n ≤ 4). As the molar ratio of x:y increased to 2:8, excitonic absorption peak intensity at 430, 462, and 478 nm is slightly enhanced, corresponding to the n phases of 2, 3, and 4, respectively. With the increasing concentration of EOABr, the excitonic absorption peaks of large-n phases (n ≥ 5) around 503 nm show a minor blue shift. Meanwhile, gradually blue-shifted PL spectra were also found from 508, 507 to 505 nm with the increased EOABr molar ratio (Figure 1B). These trends can be interpreted by the increase of n ≤ 4 phases together with the reduction of n ≥ 5 phases with more EOABr added.

**FIGURE 2:**
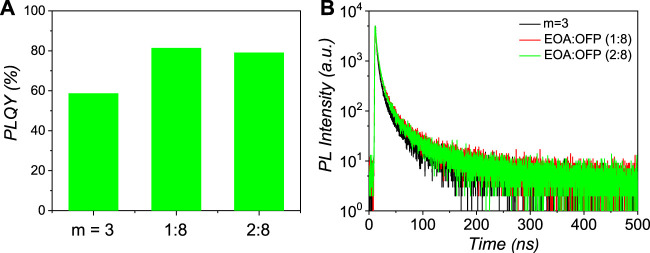
**(A)** PLQY and **(B)** time-resolved PL decay curves of the corresponding Q-2D perovskite films with different interlayers.

PLQYs of these samples are shown in Figure 2A. The PLQYs of 1:8 and 2:8 samples are 81.4 and 79.1%, respectively, which are much larger than that of m = 3 perovskite. Figure 2B shows the corresponding time-resolved PL decay kinetics spectra. The PL decay curves are fit with a tri-exponential decay model (Supplementary Table S3) (Sun et al., 2016; Yang et al., 2017; Li et al., 2018). The 1:8 perovskites show the highest average PL lifetime (τ_ave_) of 57.6 ns, which is larger than that of m = 3 (47.1 ns) perovskites, demonstrating that excitons in the mix-interlayer perovskites can live for a longer time. Supplementary Equations S1, S2 in the Supporting Information are used to calculate the radiative (k_r_) and non-radiative (k_nr_) transition rates. The m = 3 perovskite shows a moderate k_r_:k_nr_ ratio of 1.43. The 1:8 and 2:8 perovskites show significantly increased k_r_:k_nr_ ratios of 4.36 and 3.78, respectively. Therefore, non-radiative channels in mix-interlayer Q-2D perovskites are suppressed through more effective interlayer ligand passivation.

The X-ray diffraction (XRD) test was carried out to analyze the crystal structures of these mix-interlayer perovskites (Supplementary Figure S5). The diffraction pattern of CsPbBr_3_ (stand PDF# 18-0364) was also included for comparison. The 2θ diffraction peaks at 15.24° and 30.71° are observed from all the perovskite films, which ascribe to the (100) and (200) planes of the large-n phase (n ≥ 5) perovskite similar to CsPbBr_3_. The stronger XRD peak intensity of 1:8 and 2:8 samples indicated more dominant crystal orientation.

The film morphology of these samples was examined using SEM (Supplementary Figure S6). The SEM images show smoother film morphology with the addition of EOABr. The high-quality perovskite films of (EOA _x_ o-F-PEA _y_)_2_Cs_2_Pb_3_Br_10_ can restrain leakage current and facilitate the realization of highly efficient PeLEDs.

**FIGURE 3:**
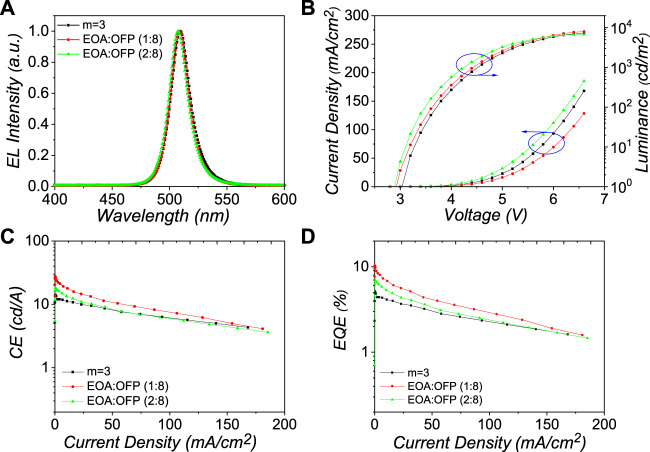
**(A)** EL spectra at 1 mA cm^−2^, **(B)** current density (J) and luminance (L) vs voltage, **(C)** current efficiency (CE) vs current density, and **(D)** external quantum efficiency (EQE) vs current density characteristics of the devices based on the corresponding Q-2D perovskite films with different interlayers.

Encouraged by the excellent optical and physical properties, the performance of these mix-interlayer perovskites was tested with the same device configuration of ITO (100 nm)/PVK:TPD (30 nm)/perovskite emitters (60 nm)/TPBI (35 nm)/LiF (1 nm)/Al (150 nm). Figure 3 shows the electroluminescence (EL) performance of these PeLEDs. All the devices show green emission with a single peak. The EL spectra exhibit a small blue shift from 510 to 507 nm (Figure 3A) with the increase in the molar ratio of EOABr ligand, which are well in keeping with that of the PL spectra. The FWHM value of mix-interlayer PeLEDs is about 19 nm, which shows excellent color purity.

The energy levels of perovskite emitters are exhibited in Supplementary Figure S7. The valence band of m = 3, 1:8, 2:8 is 5.47, 5.46, and 5.45 eV, respectively. Subtraction by the optical bandgaps gives the corresponding valence band of 3.09, 3.07, and 3.05 eV, respectively. Thus, efficient carrier injection between transporting layers and perovskite emitter layers could be achieved. The lowest unoccupied molecular orbital of PVK:TPD (−2.3 eV) can confine the electron current within the perovskite emitting layers. The hole current can also be restricted within the perovskite emitting layers via the deep highest occupied molecular orbital of TPBI (−6.2 eV). Furthermore, this device structure can realize effective carrier injection and confinement in perovskite emitting layers. Figure 3B shows the current density (J)–voltage (V)–luminance (L) spectra. Here, the decreased V_on_ of mix-interlayer perovskites comes from the slightly reduced hole carrier injection barrier.

All the EL performance parameters are summarized in Table 1 and Supplementary Table S3. With the employment of EOABr, the device performance is significantly improved (Figures 3C,D). At the ratio of 1:8, the PeLEDs show best performance with peak CE and EQE values of 27.7 cd A^−1^ and 10.4%, respectively. The improved EQE can be explained by the higher PLQY, larger k_r_:k_nr_, and better film quality owing to the mix-interlayer ligands.

**TABLE 1:** Summary of the device performance with different interlayers as the light-emitting layers.

Light-emitting layers	V_on_ (V)	*CE*_*max*_ (cd/A)	*EQE*_*max*_ (%)	*L*_max_ (cd/m^2^)	Peak (nm)
m = 3	3.0	14.2	5.1	7,290	510
EOA:OFP (1:8)	2.9	27.7	10.4	8,000	509
EOA:OFP (2:8)	2.9	18.3	7.0	6800	507

**FIGURE 4:**
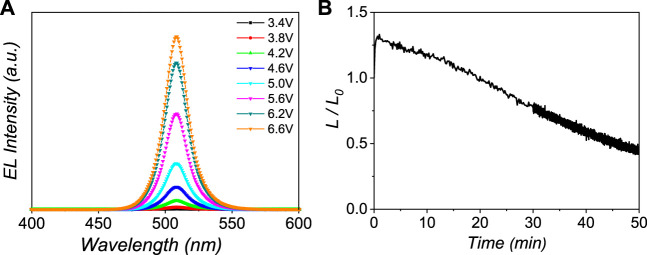
**(A)** EL spectra of EOA:OFP (1:8) PeLEDs with the operating voltage from 3.4 to 6.6 V and **(B)** T_50_ lifetime measurements for EOA:OFP (1:8) PeLEDs at an initial luminance of 100 cd m^−2^.

The device stability is one of the key parameters of PeLEDs. The spectra stability of 1:8 PeLEDs was first studied to investigate the device stability. As shown in Figure 4A and Supplementary Figure S9, all PeLEDs exhibit good spectra stability as the driving voltage increased from 3.4 to 6.6 V. In addition, the long-term device lifetime was also measured. The device lifetime was carried out in constant current mode with an initial luminance of about 100 cd/m^2^. The half-lifetime (T_50_) amounts to the time it takes to decrease the luminance to 50% of its initial value. As shown in Figure 4B and Supplementary Figure S10, the T_50_ lifetime of the m = 3, 1:8, and 2:8 PeLEDs is 34.6, 48.5, and 7.2 min, respectively. The good device stability of 1:8 PeLEDs is probably due to the better film quality, matched energy levels, and efficient carrier injection. Statistical EQE performance of the 1:8 perovskite for 30 devices is shown in Supplementary Figure S8. The average EQE was 9.1% with a relative standard deviation of 9.5%. Therefore, the 1:8 PeLEDs also show good reproducibility.

## Conclusions

In summary, efficient green PeLEDs have been realized by introducing o-F-PEABr and EOABr as the mix-interlayer ligands. The phase compositions, defect passivation, and film quality can be easily adjusted through composition and mix-interlayer engineering. Under the optimization of optical and electric properties, efficient green PeLEDs based on 1:8 mix-interlayers were obtained with a peak CE of 27.7 cd/A, a maximum EQE of 10.4%, and a T_50_ lifetime of 48.5 min. This work can provide a simple and feasible strategy for improving the performance of Q-2D PeLEDs.

## Experimental Section

**Materials**. Cesium bromide (CsBr, 99.5%), lead bromide (PbBr_2_, 99.5%), o-F-PEABr (99.5%), TPD (99.5%), and EOABr (99.5%) were purchased from Xi’an p-OLED Corp. Dimethyl sulfoxide (DMSO, 99.8%) was purchased from Acros, PVK (average Mw ∼1100000) was purchased from Sigma-Aldrich, and TPBI (99.5%) was purchased from Luminescence Technology Corp. All materials were used as received without further purification.

### Preparation of Perovskite Precursor Solution

The o-F-PEABr Q-2D perovskite precursor solution of m = 1, 2, 3, and 4 was prepared by dissolving o-F-PEABr, CsBr, and PbBr_2_ in the molar ratios of 2:0:1, 2:2.4:2, 2:3.6:3, and 2:4.8:4 in DMSO under continuous stirring for 6 h at 50°C, respectively. The mix-interlayer Q-2D perovskite precursor solution of 1:8 and 2:8 was prepared by dissolving o-F-PEABr, EOABr, CsBr, and PbBr_2_ in the molar ratios of 1.78:0.22:3.6:3 and 1.6:0.4:3.6:3 in DMSO under continuous stirring for 6 h at 50°C, respectively. The Pb^2+^ concentration in perovskite precursor solution is 0.15 M.

### Fabrication and Characterization of PeLEDs

The PeLEDs were fabricated following a well-established procedure. First, the ITO substrates were ultrasonically cleaned with detergent and deionized water. After baking at 120°C, the ITO substrates were treated with UV-O_3_ for 30 min and transferred into a nitrogen-filled glove box. 30 nm of PVK:TPD (4:1, w/w, chlorobenzene) was spin-coated and then baked at 130°C for 20 min. 1,4-Dioxane was spin-coated onto PVK:TPD at 3,000 rpm to increase the surface wettability. Then, perovskite emitters were spin-coated from the precursor solution and annealed at 95^o^C for 20 s. Finally, TPBI (35 nm), LiF (1.0 nm), and Al (150 nm) were evaporated under a pressure of 1 × 10^–4^ Pa. The thickness of the evaporated materials was monitored by a quartz crystal thickness monitor (SQM-160, Inficon). Deposition rates of TPBI, LiF, and Al were 1.5 Å s^−1^, 0.1 Å s^−1^, and 4 Å s^−1^, respectively. The emission area of these PeLEDs was 2 mm * 2 mm. All the fabrication processes were accomplished inside a nitrogen dry box. The oxygen and moisture of nitrogen dry box were less than 1 ppm. Current density (J)–voltage (V) curves were measured by using a dual-channel Keithley 2400 instrument. The EL spectra, CE, and EQEs were measured by using an integrating sphere, a multi-channel analyzer PMA-12, and an external quantum efficiency measurement system (C9920-12, Hamamatsu Photonics, Japan) (Mo et al., 2016). Before the measurement in the atmosphere, all the devices were encapsulated with a UV-cured epoxy resin.

### Measurement and Characterization

UV–Vis absorption spectra were collected by a SHIMADZU/UV-3600 PLUS spectrophotometer. XRD spectra were measured by a multipurpose X’Pert PRO system. SEM images were taken with a ZEISS/SIGMA500 system. PL spectra were measured using an Edinburgh FL980 fluorescence spectrophotometer with a 375 nm xenon lamp as the excitation light source. Time-resolved PL decay spectra were measured with an Edinburgh FL980 fluorescence spectrophotometer with a 371.6 nm ps diode laser as the excitation light source. PLQYs of the perovskite films were measured by a commercialized PLQY measurement system from Ocean Optics with a 375 nm LED as the excitation light source. The energy level values were measured by atmospheric ultraviolet photoelectron spectroscopy (Riken Keiki AC-3).

## Data Availability

The original contributions presented in the study are included in the article/Supplementary Material, and further inquiries can be directed to the corresponding authors.
