# Supplementary material for: Efficient Green Quasi-Two-Dimensional Perovskite Light-Emitting Diodes Based on Mix-Interlayer
Source: Front Chem. 2022 Jan 17;9:825822. doi: 10.3389/fchem.2021.825822 (PMC8802909; doi:10.3389/fchem.2021.825822)
Supplement: Supplementary file 1 [file DataSheet1.docx]

**Supplementary Information**

**Efficient Green Quasi-Two-Dimensional Perovskite Light-Emitting Diodes Based on Mix-Interlayer**

Zirong Wang,^a^ Fanyuan Meng,^a^* Qi Feng,^a^ Shengxuan Shi,^a^ Langwen Qiu,^a^ Guanwei Sun,^c^ Zhao Chen,^a^* Qingguang Zeng,^a^* Weiguo Zhu,^b^ Shijian Su^c^

*^a^* School of Applied Physics and Materials, Wuyi University, Jiangmen 529020, China.

* Corresponding author. E-mail: mfybys@163.com; chenzhao2006@163.com

zengqg1979@126.com

*^b^* Jiangsu Engineering Laboratory of Light-Electricity-Heat Energy-Converting Materials and Applications, Changzhou University, Changzhou 213164, China.

*^c^* State Key Laboratory of Luminescent Materials and Devices, Institute of Polymer Optoelectronic Materials and Devices, South China University of Technology, Wushan Road 381, Guangzhou 510640, China

Figure S1. (a) EL spectra at 1 mA cm^-2^, (b) current density and luminance vs. voltage, (c) current efficiency (CE) vs. current density, (d) external quantum efficiency (EQE) vs. current density characteristics of the devices based on the corresponding Q-2D perovskite films with different m values.


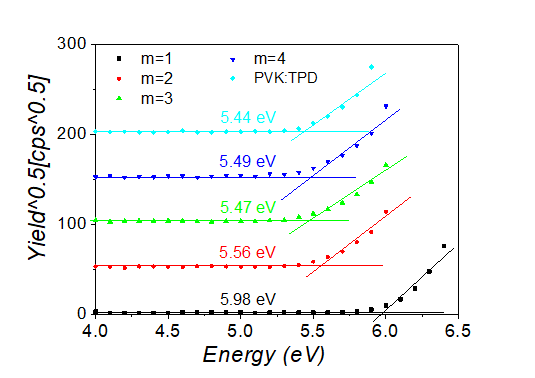
Figure S2. Atmospheric ultraviolet photoelectron spectroscopies of the HTL and Q-2D perovskite films with different m values.

Figure S3. (a) UV-vis absorption spectra, (b) PLQY of the corresponding Q-2D perovskite films with different m values.

Figure S4. SEM images of the corresponding Q-2D perovskite films with (a) m=1, (b) m=2, (c) m=3, (d) m=4.

Figure S5. XRD spectra of the corresponding Q-2D perovskite films with different compositions. The diffraction pattern of the CsPbBr_3_ (stand PDF# 18-0364) was also included for comparison.

Figure S6. SEM images of the corresponding Q-2D perovskite films with (a) EOA:OFP (1:8), (b) EOA:OFP (2:8).

Figure S7. Atmospheric ultraviolet photoelectron spectroscopies of the Q-2D perovskite films with different compositions.
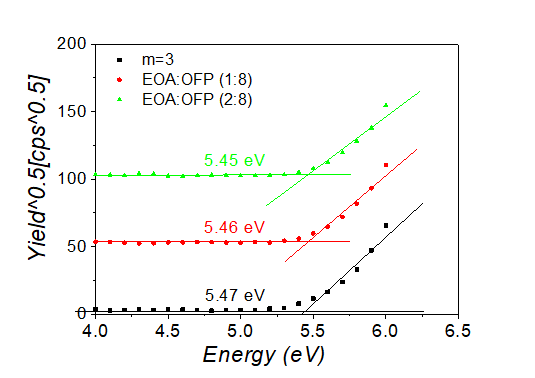


Figure S8. Statistical EQE performance of the EOA:OFP (1:8) perovskite for 30 devices. The average EQE was 9.1% with a relative standard deviation of 9.5%.
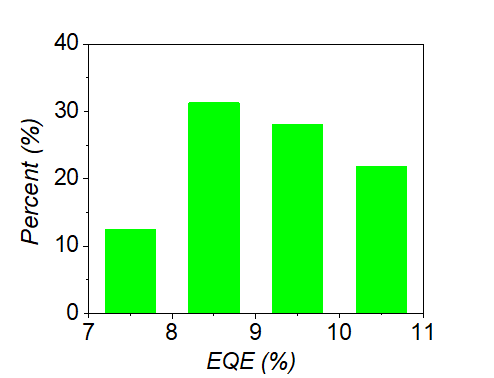

Figure S9. EL spectra of the (a) m=3, (b) EOA:OFP (2:8) PeLEDs with operating voltage from 3.4 V to 6.6 V.

Figure S10. T_50_ lifetime measurements for (a) m=3, (b) EOA:OFP (2:8) PeLEDs at an initial luminance of 100 cd m^−2^.

Table S1. Summary of the device performance of the OFPBr perovskite films with different m values as the light-emitting layers.

| Light-Emitting layers | V_on_  (V) | *CE_max_*  (cd/A) | *EQE_max_*  (%) | *L_max_*  (cd/m^2^) | Peak (nm) |
| --- | --- | --- | --- | --- | --- |
| m = 2 | 3.8 | 2.1 | 1.1 | 130 | 502 |
| m = 3 | 3.0 | 14.2 | 5.1 | 7290 | 510 |
| m = 4 | 3.2 | 3.9 | 1.4 | 2180 | 511 |

Table S2. Summary of the average lifetime (*τ_ave_*), radiative (*k_r_*) and non-radiative transition rate (*k_nr_*) of the perovskite light-emitting layers with different compositions.

| Light-Emitting-Layers | *τ_1_*  (ns) | *f_1_*  (%) | *τ_2_*  (ns) | *f_2_*  (%) | *τ_3_*  (ns) | *f_3_*  (%) | *χ^2^* | *τ_ave_*  (ns) | PLQY  (%) | *k_r_*  (10^6^ s^-1^) | *k_nr_*  (10^6^ s^-1^) | *k_r_:k_nr_* |
| --- | --- | --- | --- | --- | --- | --- | --- | --- | --- | --- | --- | --- |
| m = 3 | 2.4 | 47.0 | 12.5 | 39.3 | 70.0 | 13.8 | 1.28 | 47.1 | 58.7 | 12.4 | 8.70 | 1.43 |
| EOA:OFP (1:8) | 2.5 | 34.2 | 11.0 | 45.9 | 65.0 | 19.9 | 1.29 | 57.6 | 81.4 | 14.1 | 3.23 | 4.36 |
| EOA:OFP (2:8) | 2.7 | 37.9 | 10.4 | 44.1 | 61.0 | 18.0 | 1.30 | 43.4 | 79.1 | 18.2 | 4.82 | 3.78 |

**Calculation of radiative (*k_r_*) and non-radiative transition rate (*k_nr_*):**

*k_r_* and *k_nr_* of the perovskite films with different compositions were calculated following the Equations (S1) and (S2) below:

*k_r_ = Φ/ τ_ave_*  (S1)

*k_nr_ = 1/ τ_ave_ - k_r_* (S2)

where *Φ* represents PLQY, *τ_ave_* represents average lifetime.

**Table S3**. Summary of device performance parameters for published green Q-2D PeLEDs.

| **Light-Emitting layers** | **EL**  **(nm)** | **EQE**  **(%)** | **Anti-Solvent** | **Outcoupling** |
| --- | --- | --- | --- | --- |
| HABr-Cs_2_PbBr_5_ ^[1]^ | 520 | 1.1 | N | N |
| PEA_2_(MA)_4_Pb_5_Br_16_ ^[2]^ | 526 | 7.4 | chloroform/  toluene | N |
| PEA_2_(FAPbBr_3_)_n-1_PbBr_4_ ^[3]^ | 532 | 14.36 | chlorobenzene | N |
| BIZBr-FAPbBr_3_ ^[4]^ | 538 | 7.7 | chlorobenzene | N |
| PEABr-CsPbBr_3_ ^[5]^ | 514 | 1.97 | N | N |
| POEA-MAPbBr_3_ ^[6]^ | 506, 520 | 2.82 | N | N |
| BA_2_FA_2_Pb_3_Br_10_ (n = 3) ^[7]^ | 543 | 14.6 | chlorobenzene | N |
| BABr-MAPbBr_3_ ^[8]^ | 516, 513 | 9.3 | toluene | N |
| n-BABr-MAPbBr_3_ ^[9]^ | - | 17.5 | toluene | nanodome light couplers & nanowire optical antennas |
| EDBE(MAPbBr_3_)_n-1_PbBr_4_ ^[10]^ | 516 | 1.06 | methanol | N |
| PEA_2_Cs_n−1_Pb_n_Br_3n+1_-Crown ^[11]^ | 514 | 15.5 | N | N |
| NaBr-crown-CsPbBr_3_ ^[12]^ | 518 | 15.9 | N | N |
| PEA_2_(FAPbBr_3_)_2_PbBr_4_ ^[13]^ | ~535 | 15.4 | chlorobenzene | N |
| PEA_2_(FA_0.5_Cs_0.5_)_n−1_Pb_n_Br_3n+1_ ^[14]^ | 532 | 6.3 | chlorobenzene | N |
| F-BZABr:FAPbBr_3_ ^[15]^ | 540 | 8.55 | N | N |
| (PEA)_2_FA_2_Pb_3_Br_10_ ^[16]^ | 525 | 10.6 | isopropanol (PEABr postprocessing) | N |
| PEABr-PEG-CsPbBr_3_ ^[17]^ | 514 | 28.2 | N | moth‐eye nanostructures & half‐ball lens |
| p-FPEA- CF_3_KO_3_S- MAPbBr_3_^[18]^ | 526 | 20.36 | chlorobenzene | N |
| PEABr-TFPPO- CsPbBr_3_^[19]^ | 517 | 25.6 | chloroform-TFPPO | N |
| EOA_x_ o-F-PEA_y_)_2_Cs_2_Pb_3_Br_10_^[our work]^ | 509 | 10.4 | N | N |

**References:**

[1] C. Qin, T. Matsushima, A. S. D. Sandanayaka, Y. Tsuchiya, C. Adachi. Centrifugal-Coated Quasi-Two-Dimensional Perovskite CsPb2Br5 Films for Efficient and Stable Light-Emitting Diodes. *J. Phys. Chem. Lett.* **2017**, *8*, 5415.

[2] L. N. Quan, Y. Zhao, F. P. Garcia de Arquer, R. Sabatini, G. Walters, O. Voznyy, R. Comin, Y. Li, J. Z. Fan, H. Tan, J. Pan, M. Yuan, O. M. Bakr, Z. Lu, D. H. Kim, E. H. Sargent. Tailoring the energy landscape in quasi-2D halide perovskites enables efficient green-light emission. *Nano Lett.* **2017**, *17*, 3701.

[3] X. Yang, X. Zhang, J. Deng, Z. Chu, Q. Jiang, J. Meng, P. Wang, L. Zhang, Z. Yin, J. You. Efficient green light-emitting diodes based on quasi-two-dimensional composition and phase engineered perovskite with surface passivation. *Nat. Commun.* **2018**, *9*, 570.

[4] M. Yu, C. Yi, N. Wang, L. Zhang, R. Zou, Y. Tong, H. Chen, Y. Cao, Y. He, Y. Wang, M. Xu, Y. Liu, Y. Jin, W. Huang, J. Wang. Control of Barrier Width in Perovskite Multiple Quantum Wells for High Performance Green Light–Emitting Diodes. *Adv. Opt. Mater.* **2019**, *7,* 1801575.

[5] Y. F. Ng, S. A. Kulkarni, S. Parida, N. F. Jamaludin, N. Yantara, A. Bruno, C. Soci, S. Mhaisalkar, N. Mathews. Highly efficient Cs-based perovskite light-emitting diodes enabled by energy funnelling. *Chem. Commun.* **2017**, *53*, 12004.

[6] Z. Chen, C. Zhang, X. F. Jiang, M. Liu, R. Xia, T. Shi, D. Chen, Q. Xue, Y. J. Zhao, S. Su, H. L. Yip, Y. Cao. High‐performance color‐tunable perovskite light emitting devices through structural modulation from bulk to layered film. *Adv. Mater.* **2017**, *29*, 1603157.

[7] S. Lee, D. B. Kim, I. Hamilton, M. Daboczi, Y. S. Nam, B. R. Lee, B. Zhao, C. H. Jang, R. H. Friend, J. S. Kim, M. H. Song. Control of interface defects for efficient and stable quasi‐2D Perovskite light‐emitting diodes using nickel oxide hole injection layer. *Adv. Sci.* **2018**, *5*, 1801350.

[8] Z. Xiao, R. A. Kerner, L. Zhao, N. L. Tran, K. M. Lee, T.-W. Koh, G. D. Scholes, B. P. Rand. Efficient perovskite light-emitting diodes featuring nanometre-sized crystallites. *Nat. Photon.* **2017**, *11*, 108.

[9] Q. Zhang, M. M. Tavakoli, L. Gu, D. Zhang, L. Tang, Y. Gao, J. Guo, Y. Lin, S. F. Leung, S. Poddar, Y. Fu, Z. Fan. Efficient metal halide perovskite light-emitting diodes with significantly improved light extraction on nanophotonic substrates. *Nat. Commun.* **2019**, *10*, 727.

[10] C.-H. Chen, Z. Li, Q. Xue, Y.-A. Su, C.-C. Lee, H.-L. Yip, W.-C. Chen, C.-C. Chueh. Engineering of perovskite light-emitting diodes based on quasi-2D perovskites formed by diamine cations. *Org. Electron.* **2019**, *75*, 105400.

[11] M. Ban, Y. Zou, J. P. H. Rivett, Y. Yang, T. H. Thomas, Y. Tan, T. Song, X. Gao, D. Credgington, F. Deschler, H. Sirringhaus, B. Sun. Solution-processed perovskite light emitting diodes with efficiency exceeding 15% through additive-controlled nanostructure tailoring. *Nat. Commun.* **2018**, *9*, 3892.

[12] C. Wu, T. Wu, Y. Yang, J. A. McLeod, Y. Wang, Y. Zou, T. Zhai, J. Li, M. Ban, T. Song, X. Gao, S. Duhm, H. Sirringhaus, B. Sun. Alternative Type Two-Dimensional–Three-Dimensional Lead Halide Perovskite with Inorganic Sodium Ions as a Spacer for High-Performance Light-Emitting Diodes. *ACS Nano* **2019**, *13*, 1645.

[13] X. Yang, Z. Chu, J. Meng, Z. Yin, X. Zhang, J. Deng, J. You. Effects of organic cations on the structure and performance of quasi-two-dimensional perovskite-based light-emitting diodes. *J. Phys. Chem. Lett.* **2019**, *10*, 2892.

[14] G. Yang, X. Liu, Y. Sun, C. Teng, Y. Wang, S. Zhang, H. Zhou. Improved current efficiency of quasi-2D multi-cation perovskite light-emitting diodes: the effect of Cs and K. *Nanoscale* **2020**, *12*, 1571.

[15] J. Yan, G. Croes, A. Fakharuddin, W. Song, P. Heremans, H. Chen, W. Qiu. Exploiting Two-Step Processed Mixed 2D/3D Perovskites for Bright Green Light Emitting Diodes. *Adv. Opt. Mater.* **2019**, *7*, 1900465.

[16] W. Bi, Q. Cui, P. Jia, X. Huang, Y. Zhong, D. Wu, Y. Tang, S. Shen, Y. Hu, Z. Lou, F. Teng, X. Liu, Y. Hou. Efficient Quasi-Two-Dimensional Perovskite Light-Emitting Diodes with Improved Multiple Quantum Well Structure. *ACS Appl. Mater. Interfaces.* **2020**, *12*, 1721.

[17] Y. Shen, L. P. Cheng, Y. Q. Li, W. Li, J. D. Chen, S. T. Lee, J. X. Tang. High‐Efficiency Perovskite Light‐Emitting Diodes with Synergetic Outcoupling Enhancement. *Adv. Mater.* **2019**, *31*, 1901517.

[18] Y. Jiang, M. Cui, S. Li, C. Sun, Y. Huang, J. Wei, L. Zhang, M. Lv, C. Qin, Y. Liu, M. Yuan. Reducing the Impact of Auger Recombination in Quasi-2D Perovskite Light-Emitting Diodes. *Nat. Commun.* **2021**, *12*, 336.

[19] D. Ma, K. Lin, Y. Dong, H. Choubisa, A. H. Proppe, D. Wu, Y.-K. Wang, B. Chen, P. Li, J. Z. Fan, F. Yuan, A. Johnston, Y. Liu, Y. Kang, Z.-H. Lu, Z. Wei, E. H. Sargent. Distribution Control Enables Efficient Reduced-Dimensional Perovskite LEDs. Nature **2021**, *599*, 594.
